# realDB: a genome and transcriptome resource for the red algae (phylum Rhodophyta)

**DOI:** 10.1093/database/bay072

**Published:** 2018-07-17

**Authors:** Fei Chen, Jiawei Zhang, Junhao Chen, Xiaojiang Li, Wei Dong, Jian Hu, Meigui Lin, Yanhui Liu, Guowei Li, Zhengjia Wang, Liangsheng Zhang

**Affiliations:** 1 State Key Laboratory of Ecological Pest Control for Fujian and Taiwan Crops, Key Laboratory of Genetics, Breeding and Multiple Utilization of Corps, Ministry of Education, Fujian Provincial Key Laboratory of Haixia Applied Plant Systems Biology, Fujian Agriculture and Forestry University, Fuzhou 350002, China; 2 State Key Laboratory of Subtropical Silviculture, School of Forestry and Biotechnology, Zhejiang Agriculture and Forestry University, Hangzhou 311300, China; 3 Biotechnology Research Center, Shandong Academy of Agricultural Sciences, Jinan 250100, China; 4 College of Life Science, Shandong Normal University, Jinan 250014, China

## Abstract

With over 6000 species in seven classes, red algae (Rhodophyta) have diverse economic, ecological, experimental and evolutionary values. However, red algae are usually absent or rare in comparative analyses because genomic information of this phylum is often under-represented in various comprehensive genome databases. To improve the accessibility to the ome data and omics tools for red algae, we provided 10 genomes and 27 transcriptomes representing all seven classes of Rhodophyta. Three genomes and 18 transcriptomes were *de novo* assembled and annotated in this project. User-friendly BLAST suit, Jbrowse tools and search system were developed for online analyses. Detailed introductions to red algae taxonomy and the sequencing status are also provided. In conclusion, realDB (realDB.algaegenome.org) provides a platform covering the most genome and transcriptome data for red algae and a suite of tools for online analyses, and will attract both red algal biologists and those working on plant ecology, evolution and development.

Database URL: http://realdb.algaegenome.org/

## Introduction

Red algae (phylum Rhodophyta) have various values in our daily life. They are important sources of food, such as nori used in sushi and pudding made of Irish moss. The high content of vitamins and proteins of red algae-derived foods has made them attractive and popular in east Asia for >1000 years (1). Red algae have valuable ecological roles, such as producing oxygen in the seawater while some species are important in the formation of tropical reefs. In many Pacific atolls, red algae have contributed far more to reef structure than other organisms including corals (2). In the oceans, various species of red algae are primary producers eaten by fish, crustaceans, worms and gastropods.

Red algae occupy the second basal branch in the green lineage following the Glaucophyta algae (3). Some red algal species have important evolutionary value for studying basic biological questions such as the origin of multi-cellularity (4), symbiosis (5) and evolution of photosynthesis. There are about 6000 species of red algae [Source: AlgaeBase (6), www.algaebase.org], ranging from single-celled species to complex, multi-cellular, ‘plant-like’ organisms. They are also excellent material to study symbiosis, since many are inexorably associated with other organisms. Some species are used to produce agars, which are gelatinous food additives and in science labs as a support substance in culture media (7).

**Figure 1.**
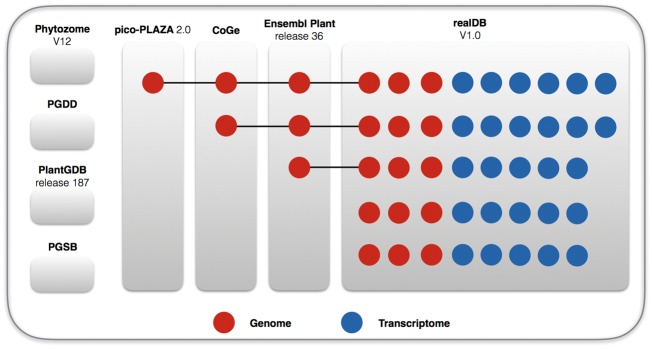
Genomes and transcriptomes included in realDB showing the comparison of datasets among several leading comprehensive databases. Phytozome V12, PGDD, PlantGDB, and PGSB have not included any red algal genome. Pico-Plaza 2.9, CoGe and Ensembl Plant each contains 1, 2, and 3 red algal genomes, respectively. In comparison, realDB now has 10 red algal genomes and 27 transcriptomes.

The current available red algae related data, such as those included in AlgaeBase (www.algaebase.org) and *Porphyra* website (http://www.porphyra.org/), are limited to morphological descriptions. The integration of genome data and morphological data is in its beginning stage. For instance, the comprehensive database phytozome V12 (phytozome.jgi.doe.gov/pz/portal.html), plant genome duplication database (PGDD, chibba.agtec.uga.edu/duplication), plant genome database (PlantGDB, plantgdb.org) (release V187) and plant genome and systems biology (PGSB, pgsb.helmholtz-muenchen.de/plant) database do not include any red algae genome. The pico-Plaza 2.0 (bioinformatics.psb.ugent.be/plaza/versions/pico-plaza/) database include one red algal genome, while CoGe database has two and Ensembl Plant database (plants.ensembl.org) has three (Figure 1). This dearth of information leads to the underestimation of the biological importance of red algae. Comparative analysis of red algae species, such as the evolutionary studies of genes families (8, 9), non-coding genes and small RNAs (10) lag far behind in plant science, partly because of the difficulty in obtaining red algae genomic information. In breeding, an open platform integrating various omics data and species information is the demand for scientists and breeders (11).

To meet the ever-rapid increasing amounts of genomic and transcriptome data and their tremendous potential in understanding developmental process (12) and to assist molecular breeding, we build an online, searchable platform for integrating the ome data with the use of multiple omics tools. The Information gained will be a valuable to boost the understanding red algae genomes and the evolution of plant genomes.

## Data description

### Dataset

Sequences from six genomes (including partial genomes) and seven transcriptomes, together with annotation data were downloaded directly from public available websites. These data were shared freely on these websites, which provided no or little online analysis tools. Raw reads of four genomes and 20 transcriptomes were downloaded from NCBI-SRA (www.ncbi.nlm.nih.gov/sra) database without annotations, thus were *de novo* assembled and annotated in this study (Supplementary Table S1). The red algal transcription factors were predicted in this study, relying on the HMMsearch tool from the HMMER software (hmmer.org) with default parameters and homology seeds from Pfam database (pfam.xfam.org).

### Assembly and annotation of genomes and transcriptomes

All the original reads from the downloaded raw data were filtered using Trimmomatic (13) (https://github.com/timflutre/trimmomatic) to remove the adapters and low-quality reads. These clean reads were then *de novo* assembled using the software Trinity (14) (https://github.com/trinityrnaseq/trinityrnaseq). Trinity produced the transcriptome files in FASTA format and the assembled sequences were then used for gene identification. TransDecoder was integrated in Trinity software and was employed for detecting gene regions (https://github.com/TransDecoder/TransDecoder/). Kyoto Encyclopedia of Genes Genomes (KEGG) and Enzyme Commission data were both obtained by BLAST genes with the KEGG database (https://www.kegg.jp/kegg/).

### Database construction

The realDB database employs Aliyun, one of the largest cloud server providers in the world, thus facilitates realDB outstanding advantages such as (i) scalability in easily expanding its storage size and computing ability, (ii) more stability and (iii) simple to maintain. The realDB relies on the Linux Ubuntu Server 14.04.4, Apache2.4.18, Java (version 1.8) and Java Server Page (JSP) 2.0. realDB provides an efficient and friendly interface for users to access a multitude of red algae data, which displays a simple and direct homepage. The searching system was created using PHP 7.0.22 and MySQL 5.7.20 software.

## Results and discussions

### An updating timeline for the sequenced red algae

**Figure 2.**
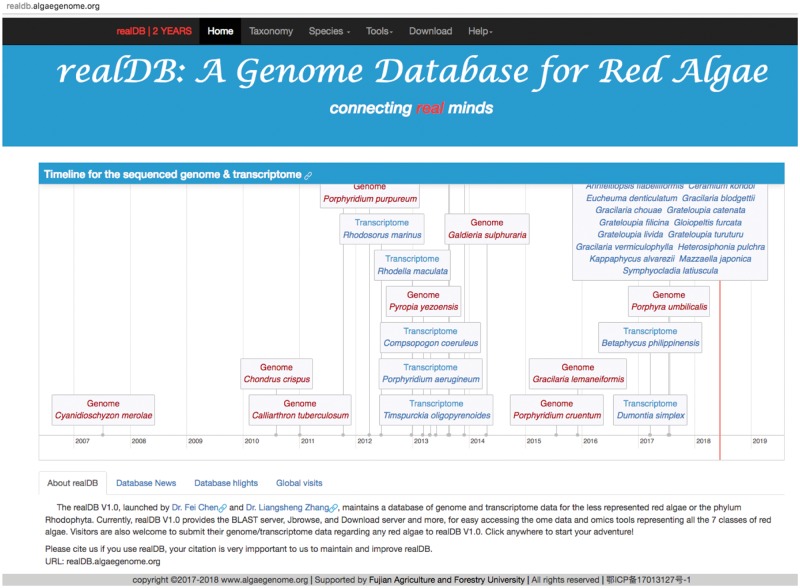
The snapshot of realDB homepage. The head part of realDB consists of two parts: the menu and the Jumbotron. A timeline was created for displaying the updates of red algal genomes and transcriptomes, together with related introduction to the sequencing of each species. realDB introduction, database news, highlights and statistics of global visits.

To attract more visits to our online platform, we created an updating timeline system on the homepage of realDB that updates the recently sequenced genome or transcriptome of red algal species (Figure 2). This timeline system consists of >1000 lines of code adapted from vis.js (http://visjs.org/), dedicated to providing multiple forms of information, including the release time, genome size, reference and authors. User can click the hyperlink to browse the reference or related linked websites for additional information. The defining feature of this timeline tool is its dynamics and interactive features with species information. Users can move the timeline space and zoom in or zoom out of the timeline by dragging and scrolling in the species timeline zone. The time-scale on the axis is adjusted automatically (http://almende.github.io/chap-links-library/graph.html), supporting scales ranging from milliseconds to years. We will create new items when genome or transcriptome from other red algal species become available.

### Bootstrap boosted framework for various display facilities

Users are able to check our genome updates and news via mobile phone using the Bootstrap framework, which is the world’s leading framework for building responsive, mobile-first sites (15). Users are able to check updates of genome releases or website news of realDB via mobile, ipad, laptop and desktop using all popular web browsers including Google Chrome, Safari, Firefox, Internet Explorer, etc. without any display difficulty (Figure 2).

### Various introductions to red algae for wide readership

The lack of red algal genome sequences in various databases is partly due to the limited knowledge of red algae. The molecular biological studies of red algae provide many useful results, such as information on systematics, physiology, ecology and evolution. Concise introductions to each species assist visitors with different backgrounds to quickly decide which species to analyze. Most of the information for each species was provided and cited from the book ‘Red Algae in the Genomic Age’ (16), including descriptions of life histories, forms and styles, genomic information and data sources. We also provided the description and classification of red algae on the website because general researchers and comparative genomic biologists usually do not have extensive knowledge of red algae classification or morphology.

### realDB covers the largest number of red algae with ome data

The current realDB V1.0 gathered 10 available genomes and 27 transcriptomes, representing all the 7 classes in the Rhodophyta. Among this dataset, we *de novo* assembled the genomes of *Galdieria phlegrea*, *Gracilariopsis lemaneiformis* and *Porphyridium cruentum*, and 18 transcriptomes (Table 1). realDB has provided 37 ome datasets, including genome and transcriptome sequences (Figure 2). In comparison, the green lineage oriented genome database Phytozome (phytozome.jgi.doe.gov) does not contain any genome/transcriptome data of red algae. Furthermore, the algae-oriented database Pico-PLAZA (bioinformatics.psb.ugent.be/plaza/versions/pico-plaza) harbors only one red algal genome, and the plant-specific database Ensemble Plant (plants.ensembl.org) has included only three red algal genomes (Figure 2). In realDB, we selected *Chondrus crispus, Cyanidioschyzon merolae*, *Galdieria sulphuraria*, *G. phlegrea* as flagship red algae with the best genome sequencing and assembly.

**Table 1.** The assembly and annotation of red algal genomes and transcriptomes in realDB

Species	Data type	Read size	Contig number	Assembled size (Mb)	Gene models	N50	Sequencing platform
*Ahnfeltiopsis flabelliformis*	Transcriptome	1.5 Gb	22 183	32.6	18 933	2748	Illumina HiSeq 2000
*Betaphycus philippinensis*	Transcriptome	1.8 Gb	23 279	28.8	15 948	2361	Illumina HiSeq 2000
*Ceramium kondoi*	Transcriptome	931.1 Mb	23 126	21.4	18 402	1385	Illumina HiSeq 2000
*Dumontia simplex*	Transcriptome	1.5 Gb	18 910	22.5	15 572	2048	Illumina HiSeq 2000
*Eucheuma denticulatum*	Transcriptome	1.7 Gb	24 656	27.9	15 478	2020	Illumina HiSeq 2000
*Gloiopeltis furcata*	Transcriptome	1.3 Gb	24 860	25.9	18 359	1594	Illumina HiSeq 2000
*Gracilaria blodgettii*	Transcriptome	735.2 Mb	19 691	22.5	15 563	2109	Illumina HiSeq 2000
*Gracilaria chouae*	Transcriptome	1.4 Gb	14 597	25.8	16 438	2904	Illumina HiSeq 2000
*Gracilaria vermiculophylla*	Transcriptome	2 Gb	13 444	25.2	15 663	3645	Illumina HiSeq 2000
*Grateloupia catenata*	Transcriptome	1.6 Gb	27 157	29	18 190	2015	Illumina HiSeq 2000
*Grateloupia filicina*	Transcriptome	1.5 Gb	49 587	38.6	25 696	1341	Illumina HiSeq 2000
*Grateloupia livida*	Transcriptome	1.3 Gb	14 934	22.2	14 131	2440	Illumina HiSeq 2000
*Grateloupia turuturu*	Transcriptome	1.4 Gb	15 739	25.5	15 639	2591	Illumina HiSeq 2000
*Heterosiphonia pulchra*	Transcriptome	1.5 Gb	33 225	28.6	19 183	1594	Illumina HiSeq 2000
*Mazzaella japonica*	Transcriptome	1.4 Gb	25 264	27	16 990	1981	Illumina HiSeq 2000
*Neosiphonia japonica*	Transcriptome	1.3 Gb	25 347	21.8	16 127	1204	Illumina HiSeq 2000
*Porphyra purpurea *	Transcriptome	869.9 Mb	20 323	24.8	655 453	1121	454 GS FLX
*Compsopogon coeruleus*	Transcriptome	1015.8 Mb	11 718	15.8	6844	2639	Illumina HiSeq 2000
*Erythrolobus madagascarensis*	Transcriptome	732.3 Mb	14 099	14.5	9152	1433	Illumina HiSeq 2000
*Erythrolobus australicus*	Transcriptome	582.5 Mb	14 227	15.4	11 857	1533	Illumina HiSeq 2000
*Kappaphycus alvarezii*	Transcriptome	1.9 Gb	34 095	40.8	20 253	1550	Illumina HiSeq 2000
*Madagascaria erythrocladiodes*	Transcriptome	1.6 Gb	51 999	48.9	39 931	1041	Illumina HiSeq 2000
*Porphyridium aerugineum*	Transcriptome	1.2 Gb	17 502	18	11 132	1450	Illumina HiSeq 2000
*Rhodosorus marinus*	Transcriptome	1 Gb	29 364	59.8	30 011	2092	Illumina HiSeq 2000
*Rhodella maculata*	Transcriptome	1.5 Gb	20 890	19.2	15 398	1434	Illumina HiSeq 2000
*Timspurckia oligopyrenoides*	Transcriptome	1.5 Gb	10 337	16.3	7826	2179	Illumina HiSeq 2000
*Symphyocladia latiuscula*	Transcriptome	939.5 Mb	32 966	22	17 377	765	Illumina HiSeq 2000
*Galdieria phlegrea*	Genome	161 Mb	11 559	13.7	10 303	1467	454 GS FLX titanium
*Porphyridium cruentum*	Genome	1.7 Gb	7321	29.3	17 005	9536	Illumina genome analyzer Iix
*Gracilaria lemaneiformis*	Genome	2.8 Gb	179 736	184	151 728	921	Illumina MiSeq
*Calliarthron tuberculosum*	Genome	1.6 Gb	119 430	99.7	28 266	718	454 GS FLX titanium
*Chondrus crispus*	Genome	1.7 Gb	925	104.8	9606	240	Sanger technology
*Cyanidioschyzon merolae*	Genome	1.8 Gb	20	15.9	5331	859 119	whole genome random sequencing
*Galdieria sulphuraria*	Genome	60 Mb	117	12	7174	134 001	ONT MinION
*Porphyra umbilicalis*	Genome	558.41 Gb	2126	85.1	14 399	202 021	PacBio RS
*Porphyridium purpureum*	Genome	7 Gb	3014	19.45	7730	20 534	Illumina GAIIx
*Pyropia yezoensis*	Genome	1.9 Gb	44 634	42.7	10 327	1669	Illumina genome analyzer Iix

### A suit of toolbox for online analysis

**Figure 3.**
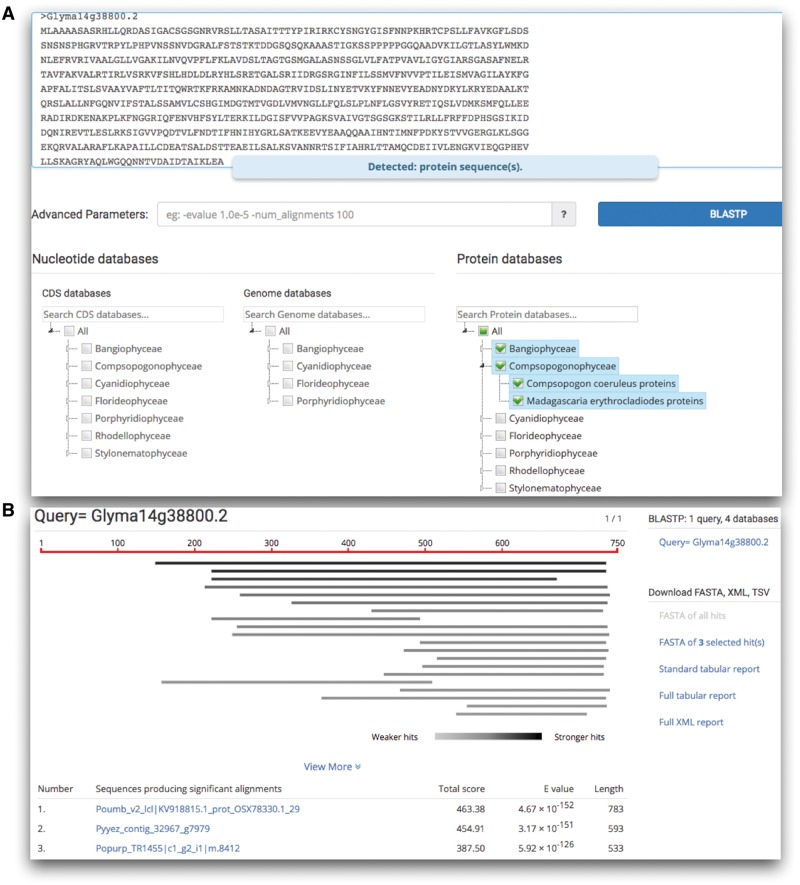
The BLAST search provided by realDB. (**A**) Users can search any combination of datasets by clicking on each red algal species. (**B**) An example of the search result. Users could download the hits in FASTA format, alignment data in tab-delimited and XML format for further analysis.

Besides the downloadable dataset, online tools would facilitate data retrieval and comparative analyses. Currently, realDB provides a complete suite of BLAST tools (Figure 3) consisting of BLASTn, BLASTx, BLASTp, tBLASTn and tBLASTx. This BLAST suit was constructed using the sequenceserver tool (www.sequenceserver.com/). A list of 21 advanced parameters such as -evalue 1.0e-10 -max_target_seqs 10 are optional for searches. For the nucleotides, coding sequences (CDS) and genomes were separated and could be individually selected. Users will find the GenBank style formatted BLAST results easy to use and download hits in FASTA format, and align data in tab-delimited or XML formats.

JBrowse was incorporated into realDB (Figure 3), allowing users to instantly browse, visualize, and retrieve sequence data. Currently, we provided the Jbrowse tool for *C. crispus*, *C*. *merolae*, *G. sulphuraria*, which are well assembled and annotated genomes. Using Jbrowse tool, users can easily browse and analyze these genomes at various scales with a graphic interface. Detailed gene information could be conveniently viewed and fetched by zooming in and out the interested genomic region, to view the information such as location, annotation and sequences by clicking on the corresponding tracks.

**Figure 4.**
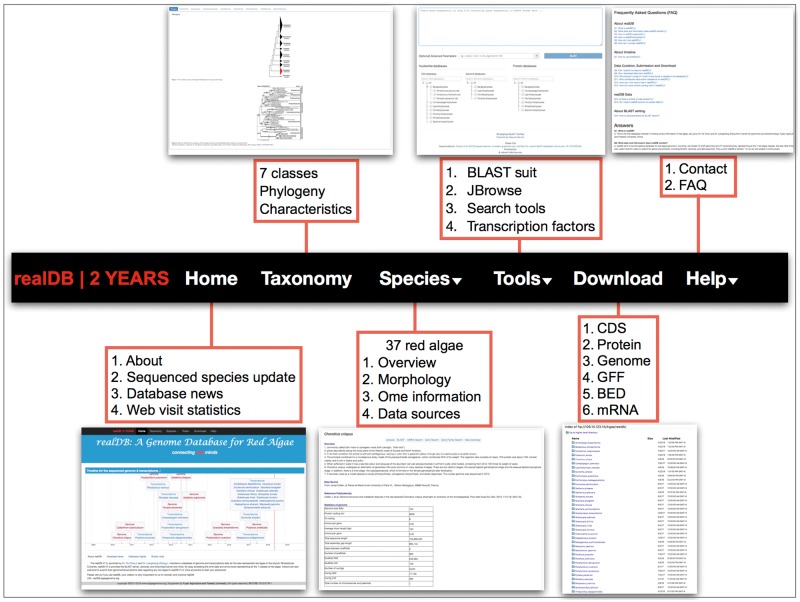
realDB offers a series of tools for online analysis. This menu offers detailed resources and tools integrated in realDB. A snapshot was presented to each menu to help readers quickly catch the related information.

The search tools in realDB provide a series of search service for CDS, protein, gene annotation, gene family, transcription factors and miRNA information (Figure 4). These information will be useful for both wet lab and dry lab biologists. Gene families, especially transcription factor families, control various physiological processes and are breeding targets (17–27). miRNAs have been extensively studied in land plants and green algae (12, 28, 29). However, little is known about its function and evolutionary trajectory in red algae. We incorporated four miRNA datasets that have been experimentally validated from *Porphyridium purpureum* (30) (Porphyridiophyaceae), *C. crispus* (31) (Florideophyaceae), *Eucheuma denticulatum* (32) (Florideophyaceae), *P. yezoensis* (33) (Bangiophyaceae) into realDB. Users can easily discover a miRNA and related information through our search system.

## Conclusion and future perspectives

Red algae (Rhodophyta) have a critical place in plant evolution as the second branch after Glaucophyta, attracting thousands of scientists in areas of ecology, evolution and genomics. They are also attractive to people working on bioengineering, medicine and food science. These study of red algae is facing the rapid development of genomics. Facilitated by low-cost and fast sequencing technologies, more and more red algae have their genomes and transcriptomes sequenced. realDB is dedicated to being the leading platform for analyzing red algae genomes by providing the latest omics data and oneline analysis tools. Currently, we provide the most genome and transcriptome data for 37 red algae that are freely available to all researchers. The realDB Version 1.0 database is the first release and will be updated when new datasets are available. Furthermore, we will incorporate additional bioinformatics tools for easier data access and online analyses. Since its release in September 2017, realDB has attracted the attention of scientists from around the world, and the website has been visited by researchers from 27 countries (April 2018). All people interested in realDB are encouraged to contact us for data sharing and collaboration. We are dedicated to collaborating with international teams to collect more data and develop more tools, hoping to make realDB the most influential database for red algae studies.

## Supplementary data

Supplementary data are available at *Database* Online.

## Supplementary Material

Supplementary Data
