# Supplementary figures and images for: realDB: a genome and transcriptome resource for the red algae (phylum Rhodophyta)
Source: Database (Oxford). 2018 Jul 17;2018:bay072. doi: 10.1093/database/bay072 (PMC6051438; doi:10.1093/database/bay072)

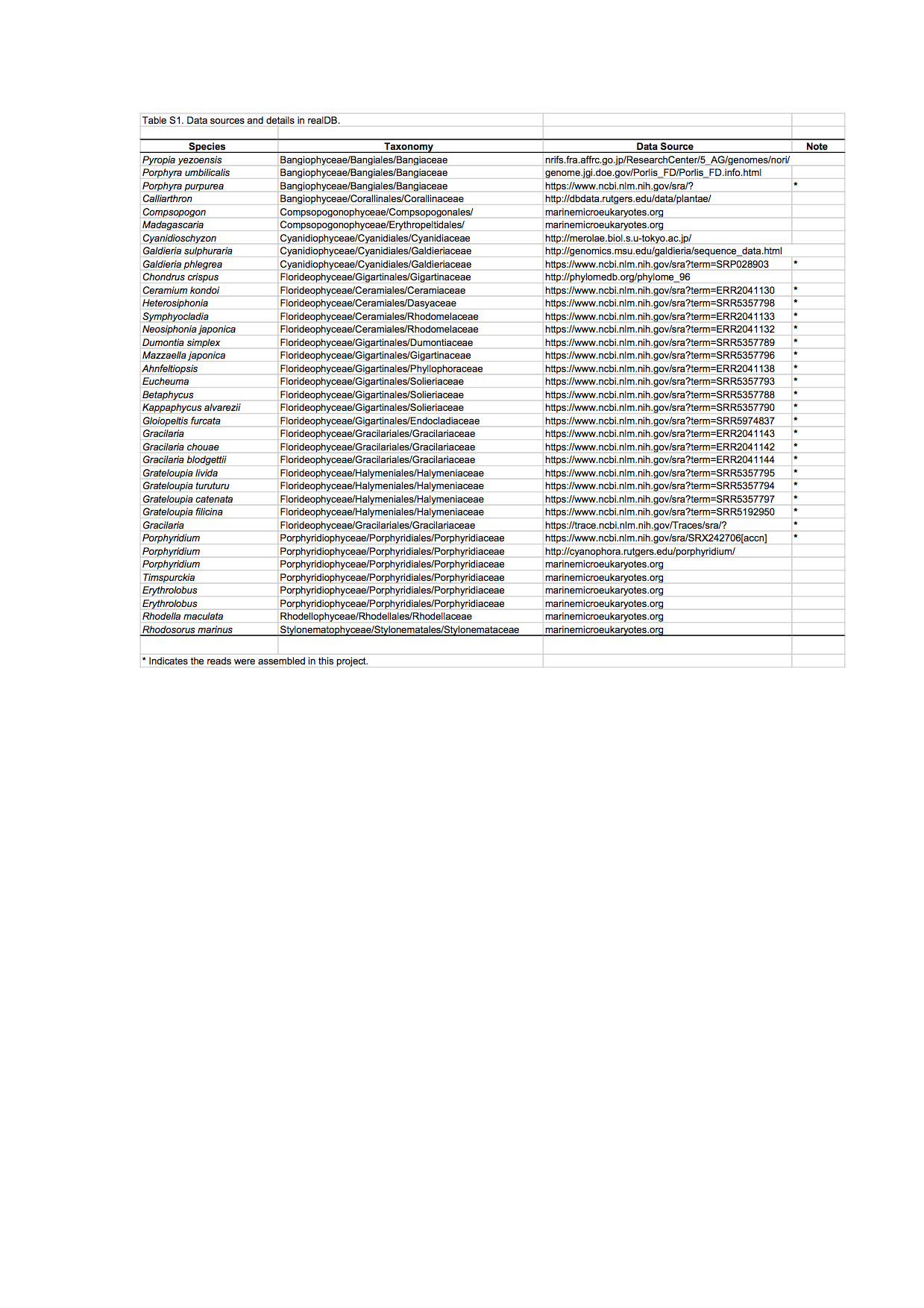

Supplement: Supplementary Data [file bay072_supp_st_1.jpeg]
